# The causes of air movement in hidden indoor micro-environments: measurements in historic bookshelves

**DOI:** 10.14324/111.444/ucloe.1976

**Published:** 2024-09-25

**Authors:** Morena Ferreira, Josep Grau-Bové, Nigel Blades, Lisa O’Hagan, Hector Altamirano

**Affiliations:** 1 UCL Institute for Sustainable Heritage, London, UK; 2 National Trust for England, Wales and Northern Ireland, London, UK; 3 UCL Institute for Environmental Design and Engineering, London, UK

**Keywords:** air movement, historic buildings, preventive conservation, micro-environment, mould

## Abstract

The use of ventilation holes in small micro-environments has been proposed by the National Trust as a mechanism to improve the environmental conditions of moisture and temperature within bookshelves. At one National Trust historic property, this mechanism has been used to encourage air movement behind books as a possible strategy to reduce the risk of mould growth. It is believed that including ventilation holes as a passive design solution to promote airflow within micro-environments could prevent decay from occurring in the archives of historic buildings. This paper investigates the mechanisms that cause airflow behind bookshelves using field measurements in three National Trust historic libraries. The measurements indicate that small but measurable velocities, up to 4 cm/s, can be passively generated behind bookshelves. Air movement in such confined micro-environments is probably caused by a combination of natural convection, caused by temperature differences between the walls and the interior and the exterior of the bookshelf, and forced convection due to drafts in the surrounding environment. While in some cases one mechanism prevailed, both mechanisms may be present simultaneously in most cases. Further research is needed to clarify how surface temperature drives air motion behind shelves.

## Introduction

Environments with higher indoor relative humidity (RH) are a considerable challenge for conservation in historic buildings as they may promote the decay of building fabric and collections [1,2]. High indoor humidity provides suitable conditions for mould development, especially in micro-environments that can occur in hidden areas of rooms, such as in corners and/or behind furniture. Higher RH and low temperatures have been found behind bookshelves in historic buildings, increasing the risk of the biodeterioration of books. It is believed that increasing air motion in shelves using ventilation holes can eliminate these conditions, which is a strategy being tested by the National Trust [3]. Such holes can have different geometries and can be put into horizontal shelves, behind the books or into the vertical backboard of the bookshelf.

It is well-known that natural convection caused by temperature (T) differences can promote ventilation in buildings [4]. This air movement mechanism has been studied extensively, but usually in geometries far larger than the micro-environments found at the back of bookshelves. Examples of spaces similar to the one studied in this paper are the ventilation cavities in walls [5], air movement within double-glazed windows [6], or air movement in the space behind paintings and walls in museums [7]. These studies have shown that mathematical modelling can successfully predict the dynamic environmental conditions of these spaces. However, micro-environmental simulations are usually compared with measurements of temperature or humidity, rather than measurements of air motion. This paper presents an alternative approach to this analysis, providing direct measurements of air velocity taken within and outside the micro-environments of interest.

This study aimed to understand which physical mechanisms are responsible for the air movement within bookshelves, using data collected in situ. This study focused on historic bookshelves in three UK National Trust properties. The selection of properties was made based on different scenarios of the incidence of mould development: a relatively constant presence of mould (Charlecote Park); some previous mould development (Blickling Hall); or no records of mould development (Ham House). This analysis resulted in an interesting dataset of monitored data that offers an insight into the behaviour of this unique micro-environment. The purpose of this paper is to present and analyse this dataset, as well as indicating which additional data future research should collect in order to reach a good understanding of the dynamic behaviour of these environments.

The environmental parameters in each property varied considerably: climate conditions, visiting patterns, the configuration of rooms and bookshelves, the orientation of rooms, etc. Therefore, the study aimed not to compare the three case studies but to use their diversity to better understand the context of air movement in such micro-environments.

## Methodology

### Monitoring locations

#### Charlecote Park

**Figure 1:**
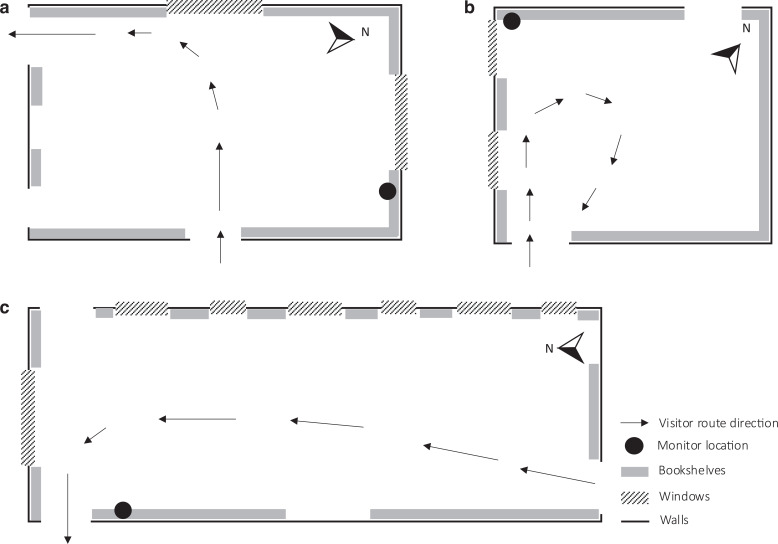
Floor plans of Charlecote Park library (a), Ham House library (b) and Blickling Hall long gallery (c) with location of the bookshelves monitored and visitor routes (approximate scale).

Charlecote Park house is a Tudor mansion built in 1558. The library and the dining room were added as a new extension to the Tudor mansion in the 1830s. The bookshelves in the library were built between 1835 and 1839 [8]. The monitored bookshelf sits next to a window in the north external wall. A second window is located on the west wall. The library is part of the visitor route. Visitors enter the room from the hallway (east entrance) and walk parallel to the wall where the monitored bookshelf is located (north-facing). However, the bookshelf is set apart from the route as visitors circulate through the centre of the room, as can be seen in Fig. 1a. Visitors are directed to the west wall and exit the room through the south wall towards the adjacent room.

#### Ham House

Ham House is a property built in the 1600s, located on the banks of the River Thames, in Ham [9]. The library at Ham House faces southwest and is situated on the first floor of the historic property. There are two doors on opposite walls (northwest where the bookshelf is located and southeast) and two windows on the southwest wall. All the walls are covered with bookshelves filled with books. Visitors use the same entrance when entering and exiting the library and do not reach the monitored bookshelf Fig. 1b.

#### Blickling Hall

Blickling Hall, in Norfolk, is a Jacobean building built in the early 1600s [10]. Its main entrance faces south. The library is in the long gallery on the first floor with windows facing east. The monitored bookshelf is on the west wall with no windows. Visitors are allowed to circulate in the library with no restricted area next to the bookshelves that cover both walls in the long room (Fig. 1c).

### Monitoring methods

For each case study, air movement, RH and T were monitored to characterise the bookshelf and the general room environments. These indoor environmental parameters were monitored simultaneously behind the books in each bookshelf and the room, in the space right next to each bookshelf. Monitors, including the anemometers used to monitor air movement, were placed at 1–2 m high to increase the chances of detecting air movement caused by visitors in the space. However, this was not possible in the case of Blickling Hall because the area is accessible by the public and the anemometer was hung on the bookshelf.

**Figure 2:**
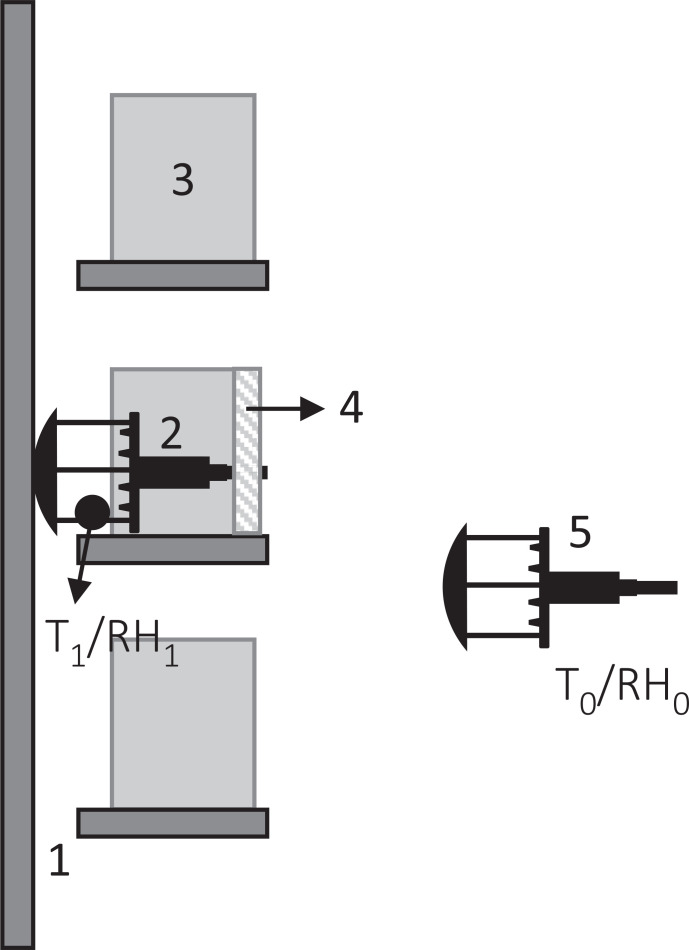
Cross section of bookshelf with gaps behind the shelves (1) anemometer installed in shelf, (2) next to books (3) and with a piece of foam covering the space taken by the anemometer, (4) temperature and RH of the shelf (T_1_/RH_1_) measured next to the anemometer; anemometer installed in the room (5) where temperature and RH was also measured (*T*_0_/*T*_0_).

Air movement (m/s) was monitored every second with two WindSonic Ultrasonic anemometers (Gill Instruments Ltd, Hampshire, UK) (accuracy: ±2% at 12 m/s; resolution: 0.01 m/s; threshold: 0.01 m/s; response time: 0.25 s; range: 0–60 m/s), each with a SpaceLogger.W8.Wireless logger (Richard Paul Russell Ltd, Hampshire, UK) to record the collected data. On shelves, the anemometer was placed against the back of the bookshelves, as seen in Fig. 2. The space taken by the anemometer, from where books were removed, was covered with a piece of foam (height 28 cm, width 17 cm), placed flush with the spine of the books to close this space and mimic the presence of books.

A second anemometer was placed in front of each bookshelf monitored to measure the air movement in the room. At Ham House, the anemometer was placed at 50 cm from the shelf, at 100 cm height (measured at the centre of the anemometer), using a tripod. The anemometer was used horizontally to measure the air movement on a vertical plane, perpendicular to the bookshelf. In Blickling Hall, the anemometer was hung from the bookshelf, at a height of 220 cm, as it was not possible to place it in the space next to the bookshelf given the visitors’ circulation. In Charlecote Park, the anemometer was placed at 106 cm from the bookshelf, at a height of 126 cm. In the three properties, T and RH were monitored within the bookshelf, next to the head of the anemometer. Equally, in each room T and RH were monitored next to the anemometer except in Blickling Hall where the monitor was placed next to the spine of the books, as seen in Table 1.

**Table 1.** Bookshelves monitored and its context in the respective historic properties, with type of gaps in shelves and monitored periods

	Charlecote Park	Ham House	Blickling Hall
Location of studied room (red dot) (north points up)	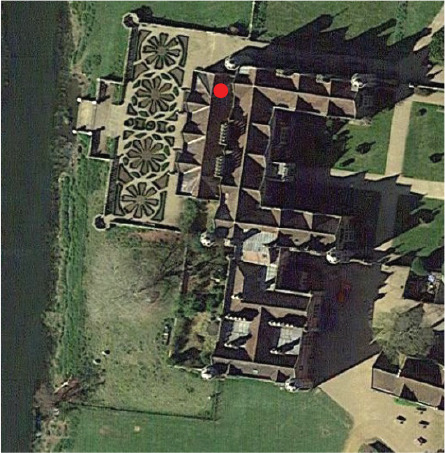	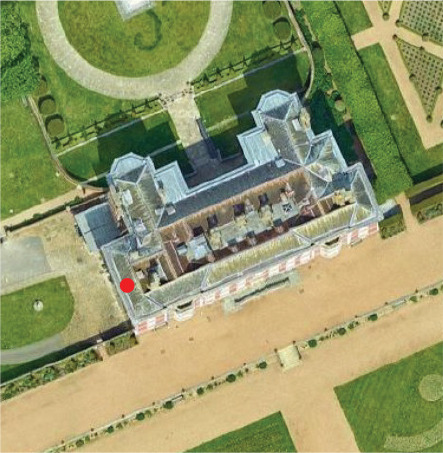	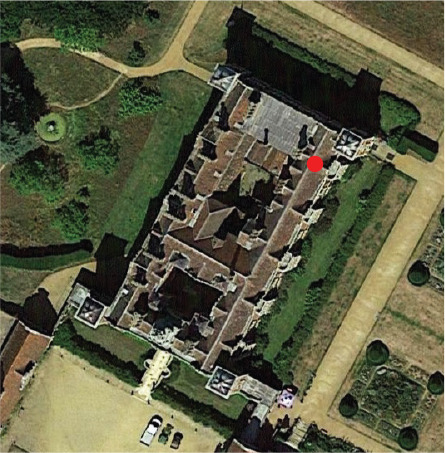
View of the shelf with anemometers in the shelf (dashed line) and in the room (circled), and T and RH monitor in the room (arrow)	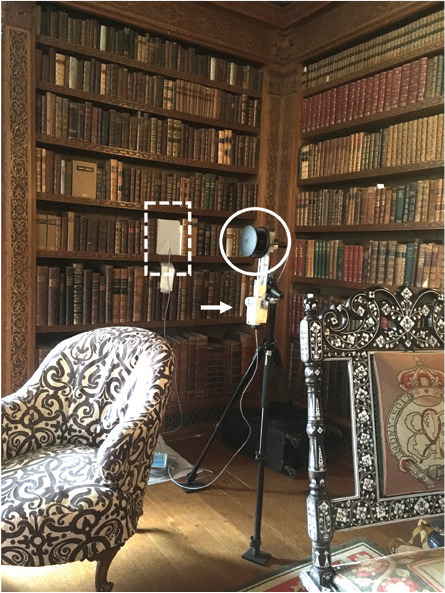	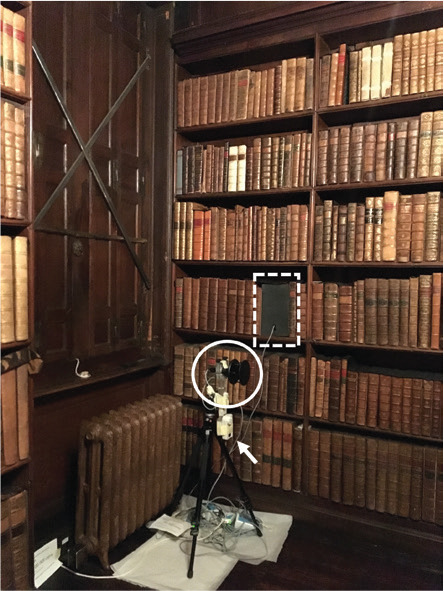	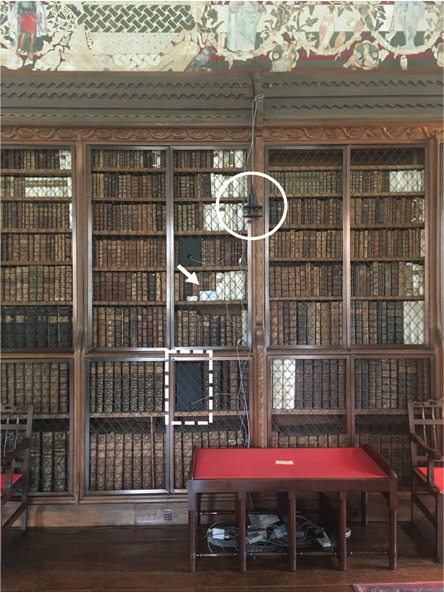
Position and dimensions of the shelf studied	149 cm wide and 27 cm high. The shelf is at 118 cm from the floor	96 cm wide, 45 cm high and 27 cm deep. The shelf is at 114 cm from the floor	40 cm high and 77 cm from the floor
Gap on the shelf	No gap	Continuous gap. 1.5 cm wide.	Continuous gap. Approximately 3 cm wide
Monitoring times	Summer: 20–24/07/19 (open to the public daily 11:30 to 16:00) Winter: 05–08/02/20 (closed to the public) and 16-20/02/20 (open to the public)	Summer: 04–08/07/19 (open to the public daily 12:00 to 16:00) Winter: 24–28/01/20 (closed to the public)	Summer: 14–18/08/19 (open to the public daily 12:00 to 17:00)
Heating in the room	Yes (conservation heating: low level wet system heating plus electric radiators on local humidistats)	Yes (conservation heating controlled by building management system)	Yes (conservation heating: wet system heating plus electric radiators on local humidistats)
Bookshelf wall	External (facing north)	Internal (facing southeast)	Internal
Visitor movement	Visitors enter the room but do not walk past the bookcase.	Visitors enter the room but do not walk past the bookcase.	Visitors enter the room and may walk in front of the bookcase.

### Modelling the stack effect

To further explore the available data, air velocities (m/s) for the monitored period were predicted using a simple model (Eq. 1), which was adapted from an equation used to calculate the draft flow rate caused by thermal forces alone [11]. While this model does not provide an exact representation of the physics of this system, it has the advantage of requiring inputs that align with the available data. If measurements of surface temperatures at the back of the shelves and the walls were available, it is preferable to explore the use of correlations for flow between isothermal flat plates, using the Rayleigh number. The advantage of the stack effect equation is that it requires the temperature inside and outside the air column of interest:

(1) $$v=C\sqrt{2gh\frac{T_{i}-T_{0}}{T_{i}},}$$

where *C* is the discharge coefficient (usually from 0.65 to 0.70), *g* is the gravitational acceleration (9.81 m/s^2^), *h* is the height or distance (m), *T_i_* is the average inside (the bookshelf) temperature (K) and *T*_0_ is the outside (in the room) air temperature (K) [11]. In buildings, *h* is taken as the height of the air column. In this case, we have used the height of the bookshelves. This is an arbitrary choice, and it could be argued that other dimensions, such as the height of an individual shelf, could also be appropriate. To estimate the discharge coefficient *C* for each of the case studies, Eq. 1 was rearranged in the shape of a linear equation of slope *k:*

(2) $$v=k\sqrt{\frac{T_{i}-T_{0}}{T_{i}}},$$

where $\;k=C\sqrt{2gh}.$ This equation is normally used in air columns, where the internal temperature is measured at the base of the column and the external temperature is measured at the top. In our case, we use the internal temperature of the shelf as *T_i_* and the external temperature as *T*_0_. With the available data this equation provides a good approximation to the driving thermal flow in this geometry. The comparison with the measurements allows a discussion on potential improvements to this simple model.

## Results

### Temperature and humidity

**Figure 3:**
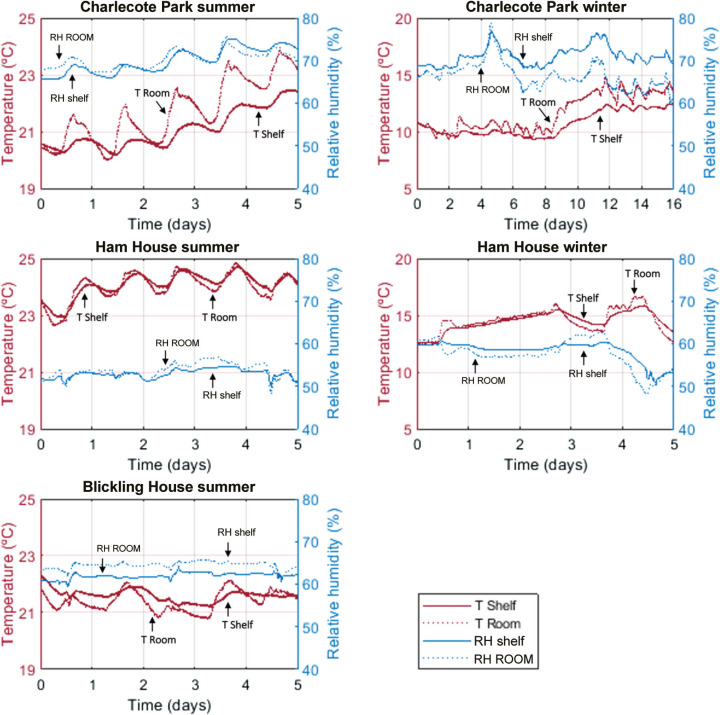
T and RH inside the bookshelves and within the rooms (vertical lines correspond to the night period, 00:00 am).

In general, the T and RH in the bookshelves follow the indoor conditions of the room, both during summer and winter. This is clearly seen in Fig. 3. The results show that shelves in general have a buffering effect [12], generating dampened fluctuation amplitudes of T and RH. This is especially clear in the T fluctuations in Charlecote Park, and the T and RH fluctuations in Ham House in the summer.

In Charlecote Park, the indoor RH recorded in the summer was mostly lower on the shelf than in the room. In the winter, we can see that the RH in the shelf is higher than in the room, possibly due to lower T influenced by the external wall. In Ham House, the room and shelf temperatures show a daily oscillation in the summer with a small delay in the T within the shelf. The RH in both are also similar, with minimal differences, which are close to the error of the equipment (±0.35 °C and ±2.5% RH). The absolute humidity inside and outside the shelf are also similar (7.27 g/m^3^ inside and 7.24 g/m^3^ outside in the winter, 11.7 g/m^3^ inside and 11.6 g/m^3^ outside in the summer). This similarity indicates that differences in RH are possibly due to differences of T, rather than the presence of sources or sinks of water. In Blickling Hall, the T and RH both in the room and the shelf are very stable, with values in the shelf slightly higher but with a smoother behaviour, which could be evidence of buffering likely due to the thermal insulation properties and hygroscopic nature of the books. In this case, there is also no significant difference between the absolute humidity inside (11.7 g/m^3^ on average) and outside (12.1 g/m^3^ on average), indicating again that temperature dictates the changes in RH.

Outdoor data of wind conditions obtained from the Met Office weather stations near the properties for the monitored periods showed no correlation with the indoor air movement measured. During the winter period, indoor conditions of RH in Charlecote Park and Ham House showed no correlation with the outdoor RH. During the summer, only the RH conditions in Charlecote Park followed the outdoor trend but with delays. Ham House and Blickling Hall showed no correlation with outdoor RH. In the winter, temperatures indoors showed no correlation with the outdoor T conditions in all three libraries. During the summer period, a correlation was observed in all three libraries, showing an influence of the outdoor T on the indoor T.

The monitored properties display a diversity of hygrothermal conditions, as seen in Fig. 3. The temperature differences between the inside and outside of the shelves are small. However, as we shall see in the following sections, it is possible to relate this data with the observed air movement.

### Air velocity in the bookshelves and the rooms

**Figure 4:**
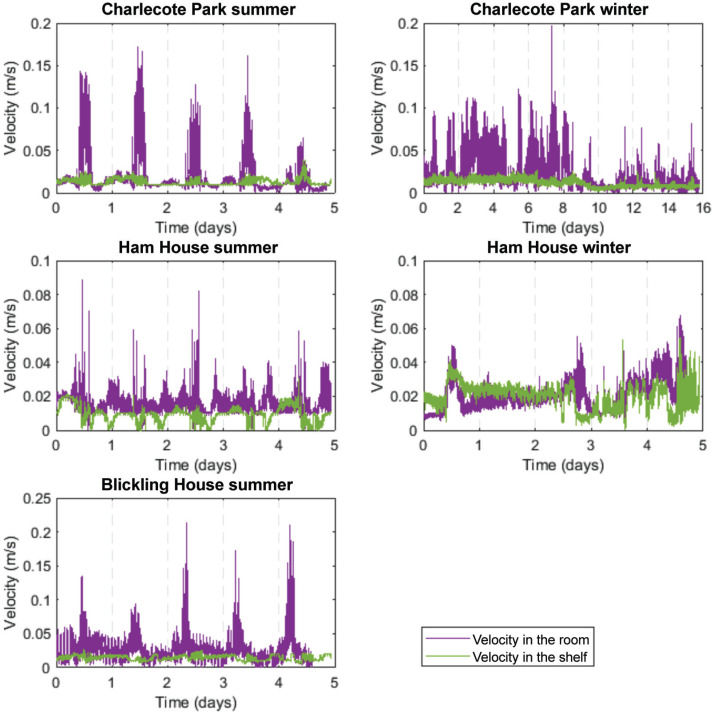
Air velocity measured inside the bookshelves and in front of them (vertical lines correspond to the night period, 00:00 am).

Air movement in the rooms usually displays a daily pattern that coincides with the opening hours of the libraries. This relationship is consistent in the three properties in the summer (Fig. 4), when the buildings are open to visitors. In these cases, air velocities in the shelf follow the changes in air velocities in the room. In the winter, the air velocity in the rooms is lower and it loses the periodicity. The air movement within the shelves is always an order of magnitude smaller than in the room, often close to the measurement threshold of the instrument (0.01 m/s).

In some properties, there is a correlation between the air movement in the room and the air movement within the shelf. This is most obvious in Charlecote Park in the summer, where air movement in the shelf is more active whenever there is air movement in the room. It can be hypothesised that the air pressure differences caused in the room either by the movement of visitors or the room’s ventilation from doors being open and closed directly affect the air movement within the shelf.

It is possible that the increase in air movement during open hours, corresponding to the peaks monitored in the room, is the result of other activities within the building; the library in Charlecote Park is adjacent to the Great Hall, where people enter the building. Thus, the increased indoor air velocities observed during visiting hours (and in the bookshelf with a smaller amplitude) could be due to general movement in the building, such as opening and closing the external doors, rather than exclusively due to people moving (walking) in the library.

During the winter period, when Charlecote Park is closed to visitors (first 4 days of monitoring: 05–08/02/20), air movement was higher both in the room and the shelf, compared with open days. This could be explained by the work carried out by staff during this period preparing the room before reopening it to the public. The increase of air movement occurred mainly around midday, coinciding with working hours and the opening and closing of the external and interior doors, similarly to the summer period.

The air movement in the Ham House and Blickling Hall rooms does not seem to be correlated with the air movement recorded in the shelves. In fact, in the summer period, the air movement in the Ham House shelf decreases when in the room it increases. In this case, the air movement in the shelf does not seem to be caused by increased air movement in the room. During this period, the property was closed to the public, but the staff were involved in works in an adjacent room and the library was used as a passageway. Their circulation in the room may explain the variation of air movement measured during this period. Therefore, air motion in the rooms alone does not explain the air movement detected within the shelves in all cases. To explore other possible causes, it is necessary to examine the differences in T between the shelf and the room.

### Air velocity and temperature differences

**Figure 5:**
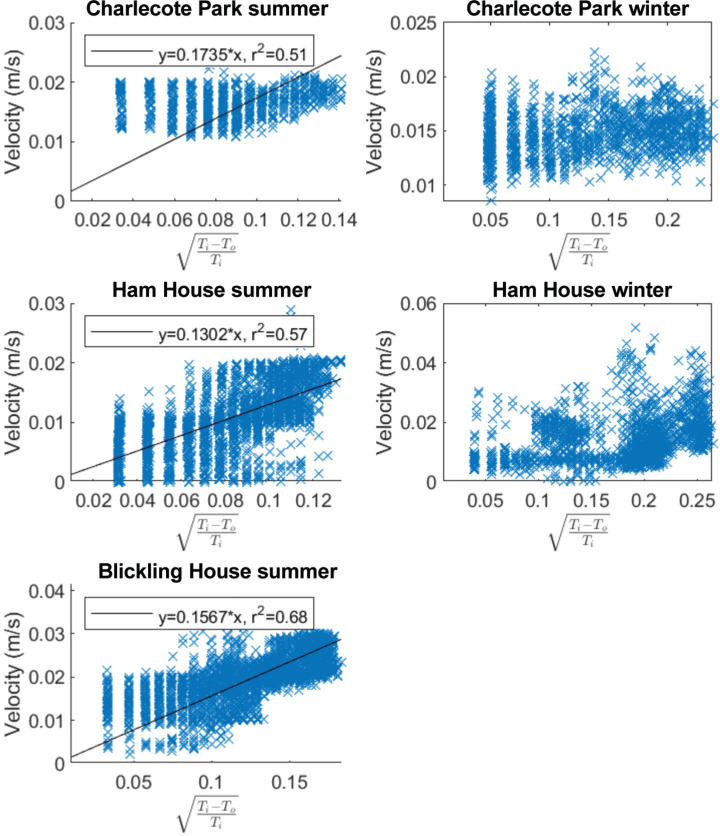
Scatterplots of velocity and the temperature difference between the interior and the exterior of the bookshelves. A linear regression line is present only in the scenarios that display a potential correlation.

As seen in Fig. 3, it is difficult to generalise about the temperatures of the bookshelves in relation to the room. The T difference changes throughout the day (between room and shelves), with most shelves experiencing periods where they are warmer or colder than the rooms. We have used Eq. 1 to explore whether these T differences are sufficient to cause the observed air movements. Figure 5 shows the relationship between the measured air velocity and T differences in each monitoring location. Note that the T differences between the rooms and the shelves are expressed in terms of $\sqrt{(T_{i}-T_{o})/T_{i}}.$ This allows the calculation of $C\sqrt{2gh}$ as the slope of the linear regression. It can be observed that in all the libraries during the summer, there is a relationship between air velocity and the T difference between the shelf and the room. While the Pearson correlation coefficients are considered moderate to strong (r^2^ ranges from 0.51 to 0.68) [13] it is evident that there exists a connection between these two variables. However, in the winter (Charlecote Park and Ham House), this relationship is non-existent, so we have not conducted the regression analysis.

Fitting a linear model to the data allows us to estimate the value of the discharge coefficient, *C*, which gives an idea of the resistance offered by the shelves to air movement resulting from stack effect. *C* is a unitless coefficient which ranges from 0 (no air flow) to 1 (no resistance to air flow).

Table 2 summarises these results. The estimated discharge coefficients are significantly lower than the value of 0.65 proposed for buildings in the American Society of Heating, Refrigerating and Air-Conditioning Engineers (ASHRAE) handbook [11], indicating a high resistance to air flow. This is to be expected given the very narrow spaces available for airflow. We found, however, no statistical relationship between these coefficients and the space available for air flow behind the shelves.

**Table 2.** Results of the estimated *C* values, together with the measured gap behind the shelves

Location	Estimate of *C*	Gap size
Charlecote Park (summer)	0.027 ± 0.000606	No gap
Ham House (summer)	0.021 ± 0.000208	Continuous gap, 1.5 cm wide
Blickling Hall (summer)	0.019 ± 0.000204	Continuous gap, 3 cm wide

**Figure 6:**
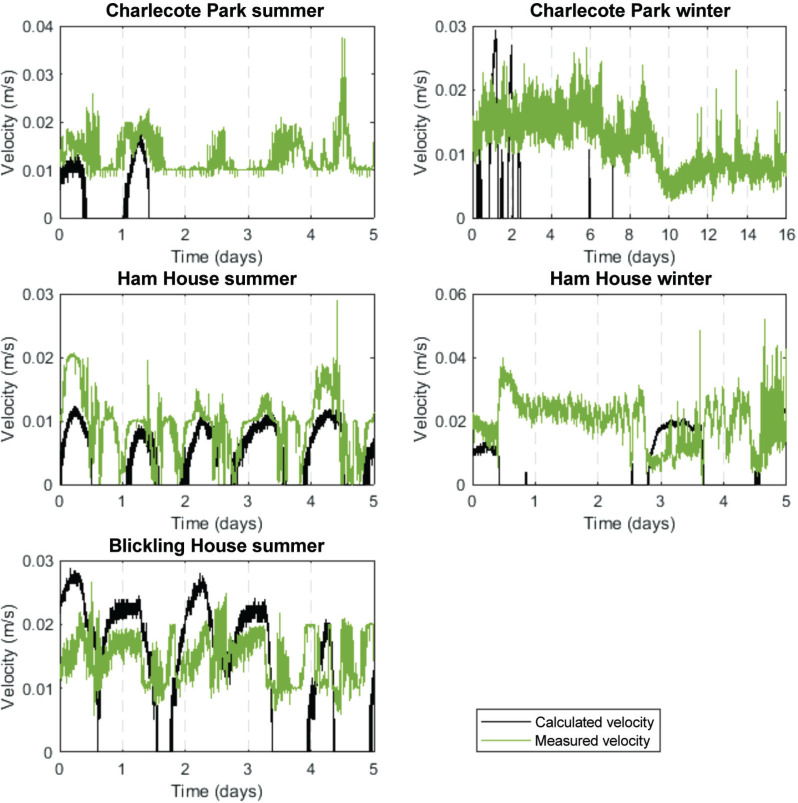
Modelled and measured air velocities behind the books (vertical lines correspond to the night period, 00:00 am).

The estimated coefficients can be used within Eq. 1 to predict air velocity. The calculated air velocity is shown below next to the measured air velocity (Fig. 6). There is a significant overlap in the data for Ham House in the summer and Blickling Hall. It is interesting to note that, in these two libraries, there is no apparent relationship between air movement in the room and within the shelves, as seen in Fig. 4. There is, however, a remarkable fit between the measured and predicted air movement in the shelves, which could indicate that air motion is caused by T differences only. This relationship is not present in Ham House and Charlecote Park in the winter, with the exception of the first 2 days in Charlecote Park in the summer, which could be explained by the activities within the house during those 2 days.

This correlation is not observed for the winter period. However, note that this does not necessarily mean there is no stack effect. It is not possible to determine if the stack effect is not present or if it is present on a smaller scale at the same time as pressure differentials have a stronger influence. It is conceivable that a pressure differential is the main mechanism responsible for the air movement monitored during the winter period, masking any potential stack effect.

## Conclusions

There are two possible explanations for the observed air movement behind books: stack effect (caused by a T differential) and pressure differentials (e.g., people circulating, opening of internal and external doors during visiting times). Forced convection caused by external pressure differences can be seen most clearly in the summer monitoring data from Charlecote Park. This property shows a correlation between the peaks of air movement measured in the room and the increase of air movement detected in the bookshelf. In this case, a pressure differential is responsible for the motion detected, very likely caused by the movement of visitors and staff members in the room. The shelves monitored in this property have no ventilation gaps. Natural convection caused by T gradients (stack effect) is visible in Ham House. This property presents a different scenario, where an increase of air velocities in the bookshelf does not coincide with the same behaviour in the room. In fact, when air movement increases in the room, the opposite happens in the bookshelf. The air movement at Ham House during this period can be explained by the T differences observed between the interior of the shelf and the room. In this property, the shelves are open behind the books with 1.5 cm continuous gaps. In Blickling Hall, using the same adapted equation, part of the air movement seems to be explained by a stack effect but not on all the monitored days. It is likely that, in reality, all cases of air motion are caused by a combination of the two processes, stack effect and pressure differences. We can recognise these behaviours more clearly when one process has a dominating effect.

The simple mathematical model used to investigate the stack effect is clearly not detailed enough to account for all the physics of the process. The main limitations are that (1) it is designed to operate with T differences over vertical columns of air, rather than differences between external and internal spaces, (2) the geometry of the shelves is more complicated than a single air column, with gaps of changing sizes and multiple openings along the way and (3) it is a linear equation which does not fully capture the actual shape of the observed relationships. Despite these limitations, the equation provides remarkably constant estimations of the discharge coefficient *C*, which are similar across the properties.

Finally, these experiments have shown that it is possible to obtain a measurable air velocity behind books on bookshelves and that gaps in shelves promote air movement. It is yet to be determined whether these low velocities are sufficient to create desirable micro-environments, for example, by inducing better ventilation which may help mitigate pockets of high humidity or cold temperatures. However, it is clear that managers in historic buildings, as well as others dealing with similar types of buildings, can take advantage of small variations in T and air velocity to promote air motion in such secluded spaces. This finding opens the door to the design of passive micro-environmental solutions.

As mentioned before, air movement can be caused by the opening and closing of doors indoors. However, several phenomena can contribute to the increase of air velocities indoors and in bookshelves, including air leakage through windows frames. Future work could explore these events to increase the understanding of air movement in bookshelves.

The question remains whether this phenomenon is sufficient to make a difference on mould growth. To explore this question, the conditions of the walls and back of the books should be examined separately. In the absence of sources or sinks of water, the absolute humidity next to these two surfaces should be the same. Exterior walls will tend to be colder and therefore have higher relative humidities. The back of books may be colder than the rest of the room, due to the external wall acting as a heat sink. In this scenario, the mild air movement detected in this research is far more likely to increase the temperature of the books than to increase the temperature of the external wall. This hypothesis should be verified by monitoring surface temperatures.

## Data Availability

The datasets generated during and/or analysed during the current study are available from the corresponding author on reasonable request.
